# Activity of fungal β-glucosidases on cellulose

**DOI:** 10.1186/s13068-020-01762-4

**Published:** 2020-07-10

**Authors:** Malene B. Keller, Trine H. Sørensen, Kristian B. R. M. Krogh, Mark Wogulis, Kim Borch, Peter Westh

**Affiliations:** 1 Department of Geosciences and Natural Resource Management, University of Copenhagen, 23 Rolighedsvej, 1958 Frederiksberg, Denmark; 2 Department of Science and Environment, Roskilde University, 1 Universitetsvej, 4000 Roskilde, Denmark; 3 Novozymes A/S, 2 Biologiens Vej, 2800 Kgs. Lyngby, Denmark; 4 Novozymes Ltd, 1445 Drew Ave, Davis, CA 95618 USA; 5 Department of Biotechnology and Biomedicine, Technical University of Denmark, 221 Søltofts Plads, 2800 Kgs. Lyngby, Denmark

**Keywords:** Beta-glucosidases (BG), Glucoside Hydrolase Family 3 (GH3), Cellulose, Enzyme specificity

## Abstract

**Background:**

Fungal beta-glucosidases (BGs) from glucoside hydrolase family 3 (GH3) are industrially important enzymes, which convert cellooligosaccharides into glucose; the end product of the cellulolytic process. They are highly active against the β-1,4 glycosidic bond in soluble substrates but typically reported to be inactive against insoluble cellulose.

**Results:**

We studied the activity of four fungal GH3 BGs on cellulose and found significant activity. At low temperatures (10 ℃), we derived the approximate kinetic parameters *k*_cat_ = 0.3 ± 0.1 s^−1^ and *K*_*M*_ = 80 ± 30 g/l for a BG from *Aspergillus fumigatus* (*Af*BG) acting on Avicel. Interestingly, this maximal turnover is higher than reported values for typical cellobiohydrolases (CBH) at this temperature and comparable to those of endoglucanases (EG). The specificity constant of *Af*GB on Avicel was only moderately lowered compared to values for EGs and CBHs.

**Conclusions:**

Overall these observations suggest a significant promiscuous side activity of the investigated GH3 BGs on insoluble cellulose. This challenges the traditional definition of a BG and supports suggestions that functional classes of cellulolytic enzymes may represent a continuum of overlapping modes of action.

## Background

Cellulases catalyze hydrolysis of the β-1,4 glycosidic bond in cellulose and are widely applied in both biorefineries and other areas including textile, laundry and paper industries [1, 2]. Cellulases are categorized in different ways. The carbohydrate-active enzymes (CAZy) database organizes glycoside hydrolases (including cellulases) on the basis of their sequence [3], and this has proven tremendously useful in many aspects of cellulase research. Some work on cellulases uses a coarser classification based on function, and distinguishes cellobiohydrolases (covering EC 3.2.1.91 and EC 3.2.1.176), endoglucanases (EC 3.2.1.4 and possibly EC 3.2.1.203) and β-glucosidases (EC 3.2.1.21). The classical definition of these three categories is that cellobiohydrolases (CBHs) attack an end (reducing or non-reducing) of a cellulose strand on the surface of the insoluble substrate. Subsequently, CBHs move processively along the strand while releasing soluble cellooligosaccharides (COS), typically with a dominance of cellobiose. Endoglucanases (EGs), by contrast, are thought to attack the strand internally and act in a non-processive way. Strictly, this would imply that the enzyme dissociated and returned to the aqueous bulk after each hydrolytic reaction, and as a result, cleaved glycosidic bonds at random positions. Finally, β-glucosidases (BGs) break down soluble COS that have been released by CBHs and EGs. Specifically, the BG cleaves off terminal pyranose units from the non-reducing end of the soluble oligosaccharides and hence produces glucose as final product of the cellulolytic process. This classification into CBH, EG and BG has proven valuable in discussions of cellulose degradation, but some results have challenged the strict distinction between these groups. For example, Cel7A from *Trichoderma reesei*, which is considered an archetypical CBH, was reported to make quite frequent endolytic attacks on crystalline cellulose [4, 5], and this would suggest a functional intermediate between CBH and EG. This mode of action is sometimes called endo-processive, and while this may represent a minor reaction path for Cel7A from *T. reesei*, other cellulases seem to use endo-processive activity as their primary mechanism [6, 7]. An intermediate mode of action was also suggested for enzymes, which are annotated as EGs. Thus, Cel5A and Cel12A from *T. reesei* showed some degree of processivity [4], and mainly produced small soluble products (glucose and cellobiose) while larger (insoluble) cellulose fragments were less frequent products [8]. These observations again point towards an endo-processive mechanism, where the EG, in contrast to the notion of random attacks, breaks the strand internally and subsequently makes a few additional cuts adjacent to the initial hydrolysis point.

Functional overlap of EGs and CBHs has been discussed for decades [5, 9], and the idea may not seem too surprising. After all, the EGs and CBHs apply the same (retaining or inverting) mechanisms on the same chemical bond, while processivity and the propensity for endo/exo attacks are probably governed by more subtle differences in the architecture of the substrate-binding region [10]. In light of this, it appears relevant to assess whether the last group, the BGs, show overlapping modes of action. In this work, we have pursued this idea by testing a group of four BGs for activity against insoluble cellulose. The kinetics and the substrate specificity of one of the BGs are further investigated. We have studied fungal enzymes from Glycoside Hydrolase family 3 (GH3), which is the family most often used for industrial breakdown of biomass [11].

### Results and discussion

**Fig. 1:**
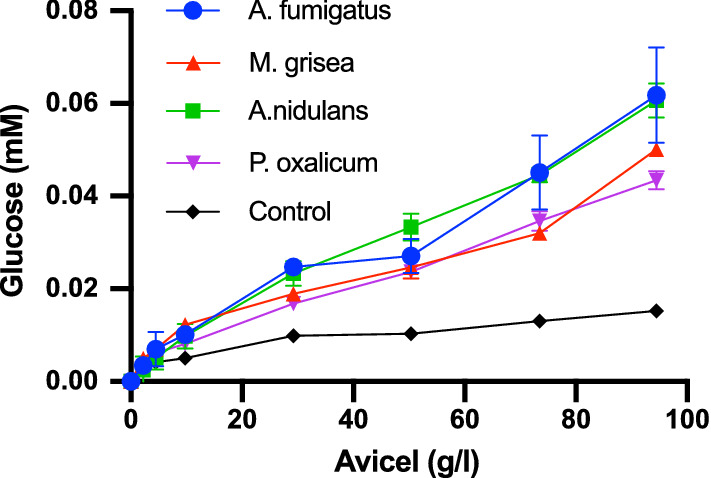
Glucose yield plotted against Avicel load for the four investigated fungal β-glucosidases. 1-h experiments at 25 ℃. Error bars represent the standard deviations (s.d.), *n *= 3. Lines are only meant to guide the eye. The controls (without BG) quantify non-enzymatic release of soluble saccharides from Avicel during the experiments (see main text for details)

*Aspergillus nidulans* BG, *Magnaporthe grisea* BG, *Penicillium oxalicum* BG, and *Aspergillus fumigatus* BG were tested for activity against microcrystalline cellulose (Avicel). The four BGs shared between 43 and 68% sequence identity (Additional file 1: Table S1). A phylogenetic tree of sequences annotated as GH3 enzymes were retrieved from the CAZy database. Only sequences annotated with β-glucosidase activity (EC 3.2.1.21) with a PDB accession were selected. Phylogenetic analysis (Additional file 1: Fig S1) showed that the four BGs studied in this work were distributed in different clades of sequences from Ascomycota. HPAEC-PAD analysis of the samples revealed that glucose was the only detectable product of all four BGs (Additional file 1: Fig. S2). Results in Fig. 1 show the glucose production from 100 nM BG as a function of the Avicel load from experiments with 1 h. contact time, and it appears that all tested enzymes had a measurable and comparable activity on this substrate.

**Fig. 2:**
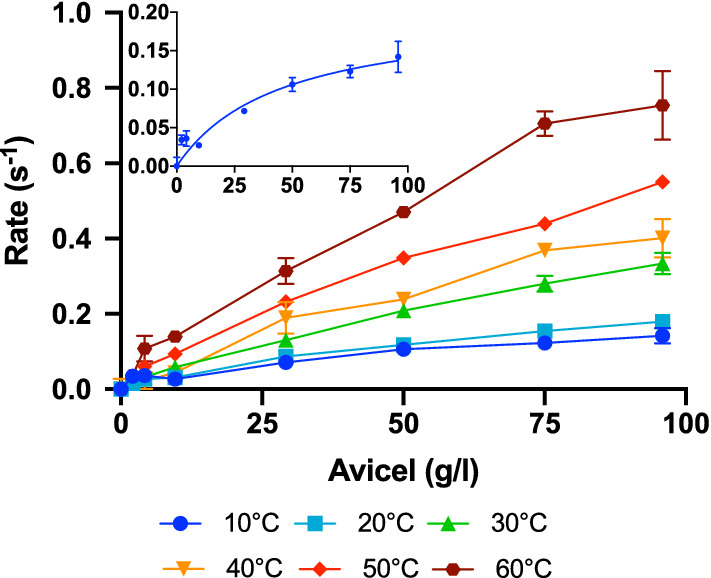
Specific rate of *A. fumigatus* BG at temperatures between 10 ℃ and 60 ℃. The activity is plotted against substrate load. For the higher temperatures, we find essentially linear relationships between Avicel load and activity, suggesting that we are far from saturation. At the lowest temperatures, we found some curvature and a fit of the Michaelis–Menten equation to the data at 10 ℃ is shown in the inset. Lines are only meant to guide the eye. Error bars represent the standard deviations (s.d.), *n *= 3

To exclude contributions from non-enzymatic release of soluble sugars (*e.g.,* due to mechanical stress in the stirred samples or extraction of restrained oligosaccharides from the cellulose matrix), we included controls with no BG for all substrate loads. The controls were incubated as the other samples and after the initial 1-h experiment, the supernatant was removed and added 100 nM *Af*BG. This would convert any soluble COS in the controls to glucose, and the black symbols in Fig. 1 indeed identified some non-enzymatic release of soluble sugars. We also made control measurements on the other cellulosic substrates and all data discussed below (and in the supporting information, Additional file 1) has been corrected by subtraction of the control value. The data in Fig. 1 may be interpreted as initial rates, and the plots hence represent Michaelis–Menten curves for the action of BG on the insoluble substrate. The near-linear relationships of glucose production and substrate load means that we cannot resolve *k*_cat_ and *K*_*M*_ at 25 ℃, and that we are far from saturation even at 100 g/l (the highest practicable load in the experiments) (*K*_*M*_ > 100 g/l at 25 ℃). To study the kinetics in more detail, we decided to zoom in on *Af*BG. First, we made a temperature series, and the results in Fig. 2 showed that at lower temperatures, the Michaelis–Menten curve got closer to saturation, and we were hence able to derive approximate values of the kinetic parameters. At 10 ℃ (inset of Fig. 2), we found, *k*_cat_ = 0.3 ± 0.1 s^−1^ and *K*_*M*_ = 80 ± 30 g/l, and these values are interesting to compare with parameters for “real cellulases”.

Typical cellobiohydrolases (Cel7A from *T. reesei* and *R. emersonii*) acting on Avicel at the same (low) temperature have values of *k*_cat_ ~ 0.02 s^−1^ and *K*_*M*_ ~ 3–5 g/l [12], and it appears that *Af*BG has faster turnover, but lower substrate affinity compared to these CBHs. We do not have Michaelis–Menten parameters for endoglucanases at 10 ℃, but at room temperature, both *k*_cat_ and *K*_*M*_ for the EG, Cel7B from *T. reesei*, are about one order of magnitude larger than the parameters for Cel7A [13]. Assuming the same ratio at 10 ℃ suggests parameters for the Cel7B of around 0.2 s^−1^ and 40 g/l, and that approximately corresponds to the values found here for *Af*BG. It may be relevant to compare the specificity constant, *η* = *k*_cat_/*K*_*M*_, which is readily measurable from the initial slope of the Michaelis–Menten curve, even in cases (as the current) where saturation cannot be reached, of the BGs with that of cellulases. Around room temperature, we found that *η* for *Af*BG was about 5 × 10^−3^ (g/l)^−1^ s^−1^(Fig. 2). This is moderately lower than specificity constants reported for the cellulases Cel6A, Cel7A and Cel7B on the same substrate. Values for these latter enzymes fall in the range (10–45) × 10^−3^ (g/l)^−1^ s^−1^ [13–16]. We conclude that the *Af*BG has kinetic hallmarks of an EG with high turnover and weak substrate binding, but that its specificity constant on Avicel is lower than for typical EGs and CBHs. This difference in *η*, however, is not large and this suggests a significant promiscuous side activity of *Af*BG on Avicel. We note in passing the unconventional (mass based) units of *η* determined on an insoluble substrate prevents comparisons with the specificity constant of *Af*BG on soluble substrates such as cellobiose.

**Fig. 3:**
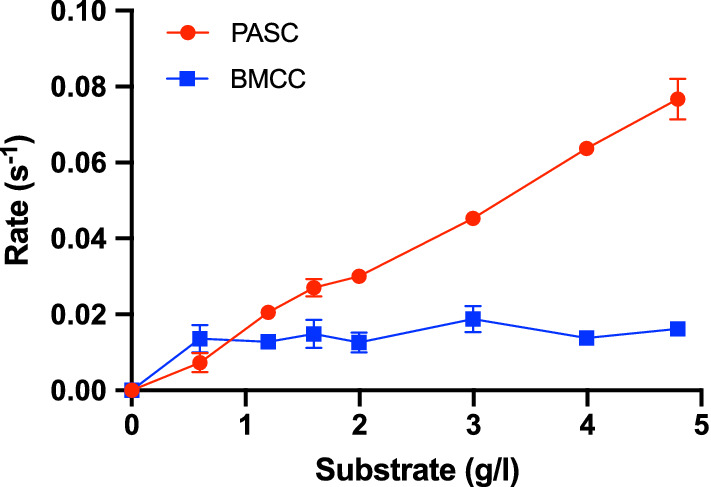
Specific rate of *A. fumigatus* BG at 25 ℃. The activity is plotted against the load of, respectively, amorphous cellulose (PASC) and bacterial microcrystalline cellulose (BMCC). Lines are only meant to guide the eye. Error bars represent the standard deviations (s.d.), *n *= 3

Figure 3 shows activity data for *Af*BG on, respectively, phosphoric acid swollen cellulose (PASC), and bacterial microcrystalline cellulose (BMCC). BMCC is highly crystalline cellulose produced by *Acetobacter xylinum*. PASC is highly amorphous and is produced by swelling microcrystalline cellulose in concentrated phosphoric acid. It appeared that the activity on the highly crystalline BMCC was very low while *Af*BG was more active on PASC. This substrate preference parallels what is typically found for EGs [10], and this again hints some functional relationship between EGs and BGs.

Earlier reports have shown that fungal BGs, have strong activity against cellooligosaccharides of different length [17–19]. Most work has focused on substrates with a degree of polymerization (DP) from 2 to 5, but in one case, it was reported that two GH3 BGs from *A. aculeatus* showed moderate activity on an essentially insoluble COS with a DP of about 20 [20]. However, the same work concluded that these enzymes were inactive on Avicel. Other works have reported that GH3 BGs showed no or very poor activity on insoluble cellulose [17, 18, 21], and the general consensus as stated in a recent review is that “fungal beta-glucosidases cannot access the insoluble cellulose fibers” [11]. The results in Figs. 1, 2, 3 suggest that this may be an oversimplification.

One possible origin of discrepancy could be that the investigated BGs were able to hydrolyze some particularly accessible structures that are initially available on Avicel such as splayed ends or other irregularities. If indeed so, one would not expect that the BG showed continued activity against Avicel in longer trials (as the hydrolysable conformations were depleted). To test this, we conducted a re-start experiment with *Af*BG, where samples prepared as in Fig. 1 were allowed to react for 17 h. The enzyme was then removed (see Materials and Methods) and a new batch of *Af*BG was added and allowed to react for 1 h. The specific rate measured for the second enzyme addition was about 60–70% of the activity over the first hour of the first addition (Additional file 1: Fig. S3). This behavior is quite typical for cellulases [22], and we conclude that there were no signs of particular limitations of *Af*BG’s activity against Avicel. Another possible reason for discrepancy with earlier literature could be impurities (particularly contamination by a cellulase) in the current samples. To assess this, we applied different purification steps and purity analyses to the *Af*BG sample. Specifically, we used an extra purification step based on size exclusion chromatography (SEC). This method was selected as the oligomeric BGs elude at a MW of about 150 kDa, which is far larger than that of known cellulases from the expression host, *A. oryzae*. *Af*BG eluted as a single, symmetrical peak in SEC, and analysis with both LabChip GXII microfluidic CE‐SDS electrophoresis (Additional file 1: Fig. S4) and filter-aided sample preparation for mass spectrometry (FASP-MS) concurrently suggested about 99% purity. The FASP-MS analysis did not identify other cellulases among the (few) impurities (Additional file 1: Table S2), and the activity of the *Af*BG sample against Avicel remained unchanged following SEC purification (Additional file 1 Fig. S5). Overall, we conclude that it is highly unlikely that impurities can account for the *Af*BG results in Figs. 1, 2, 3. Rather, we speculate that failure to acknowledge activity against cellulose in earlier work could simply rely on experimental design. Thus, BGs are typically tested against soluble substrates some of which gives rise to turnover numbers ranging into hundreds per second [17–19, 21]. At this activity level, enzymes are typically dosed in the low- or sub-nM range [21], and this may obscure activity measurements on insoluble substrates. Firstly, the product output on cellulose will be very low and hence hard to detect at this enzyme dosage. Secondly, interpretation of the results may be biased by comparisons with the rapid turnover of soluble substrates (rather than more relevant comparisons with other enzymes acting on insoluble substrates). Liu et al. [18], for example, studied two BGs from *A. fumigatus*, and concluded that they showed “very little or no activity” on Avicel and some other polymeric substrates. This conclusion was based on the observation that product formation on 1% Avicel was two orders of magnitude slower than product formation in a 1 mM solution of the soluble substrate analog 4-nitrophenyl-glucopyranoside. However, the reported activity on Avicel (about 2 U/(mg BG) at 50 ℃) corresponds to a specific activity of some 3 s^−1^. This is obviously very slow compared to the activity of typical GH3 BGs on, e.g., cellobiose [23], but it is, in fact, higher than the maximal specific rate (V_max_/E_0_) for typical CBHs [12], and in the same range as EGs [8]. In other words, if the BGs are dosed in the high nM to µM range, as it is typical for cellulases [24], they may have significant activity on Avicel.

**Fig. 4:**
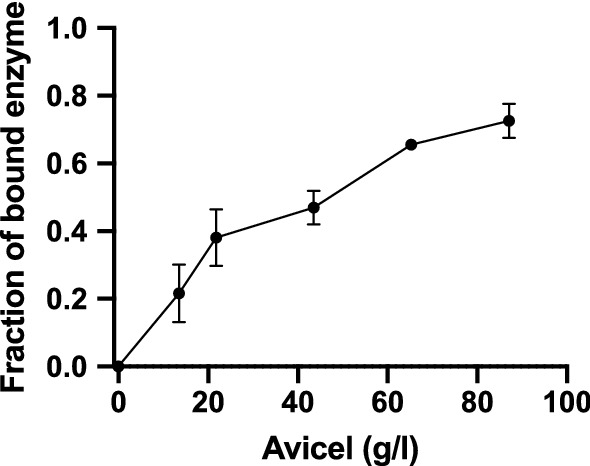
Adsorption of *A. fumigatus* BG to Avicel at 25 ℃. The fraction of bound enzyme is plotted against the Avicel load. The line is only meant to guide the eye. Error bars represent the standard deviations (s.d.), *n *= 3

We also studied adsorption of *Af*BG to cellulose. For BGs to perform heterogeneous catalysis, the BGs need to be adsorbed to the cellulose surface. Figure 4 shows the fraction of bound *Af*BG on Avicel in experiments with 100 nM *Af*BG incubated with varying Avicel loads.

The results showed that *Af*BG binds to cellulose, however, to a lesser degree than what is typically reported for CBHs [12], and this behavior parallels the observed difference in K_M_ discussed above. Due to the low adsorption of *Af*BG, we are unable to determine accurate binding constants. In order to compare the binding of *Af*BG to the binding of a typical cellulase, we have assessed the half-saturation constant, that is, the cellulose load at which 50% of the enzyme is bound. For *Af*BG that was approximately 40 g Avicel/L. Under the same conditions, the half-saturation for *Tr*Cel7A was at approximately 5 g Avicel/L, and for the *Tr*Cel7A catalytic domain without the carbohydrate binding module that is 20 g Avicel/L [12]. Adsorption of BGs to cellulose and cellulosic biomass has been reported earlier [24, 25]. However, these reports have attributed the adsorption to unspecific binding. Coupled with the activity data in Figs. 1, 2, 3, the results presented here suggest that the BGs are also productively bound, which enables them to catalyze the hydrolysis of the insoluble substrate.

Cellulases have cleft- or tunnel-shaped substrate-binding regions lined with aromatic residues that engage in stacking interactions with glucopyranose residues of the cellulose strand [10]. For *Tr*Cel7A and *Tr*Cel7B at least nine glucosyl binding subsites have been identified (− 7 to + 2) [26, 27]. GH3 BGs have pocket or crater topology [28] with only two glucosyl binding subsites (− 1 and + 1) generally described [29]. Thus, it is interesting to consider how some BGs can accommodate a cellulose chain in their active sites. Suzuki et al. [30] reported that the binding pocket of a GH3 BG from the fungus *Aspergillus aculeatus* was positioned in a groove-like structure with aromatic residues and identified additional glucosyl binding subsites (− 1 to > 5), which the authors suggested to be suitable for binding long COS [30]. Interestingly, a similar groove exists in the crystal structure of *Af*BG [31], and we speculate that this could be essential for its activity against cellulose. The crystal structures of the other BGs in this study remain to be determined.

## Conclusions

We have shown that some fungal GH3 BGs are able to hydrolyze insoluble cellulose. Kinetic parameters of *Af*BG on Avicel were comparable to those reported previously for EGs on the same substrate although the specificity constant was moderately lower. We suggest that this signifies a noteworthy side activity of some GH3 BGs on insoluble cellulose. On a practical level, this observation may call for caution in the common procedure of adding BGs to the samples in CBH assays. This is done to alleviate product inhibition under the (potentially false) premise that the BG does not act on a polymeric substrate and it may hence lead to inaccurate activity data for the CBH. Furthermore, the results challenge the traditional definition of a BG and support the view that the cellulase archetypes CBH, EG and BG represent a useful simplification of a continuum of specificities.

## Materials and methods

### Substrates

Avicel PH-101 was purchased from Sigma-Aldrich. Phosphoric acid swollen cellulose (PASC) was produced from Avicel PH-101 as described elsewhere [32]. Bacterial microcrystalline cellulose (BMCC) from *Acetobacter xylinum* was purified from the commercial, food-grade product Nata de Coco (CHAOKOH) [33].

### Enzymes

*Aspergillus nidulans* BG (UniProt: A0A2T5LVU4), *Magnaporthe grisea* BG (UniProt L7J2B9), *Penicillium oxalicum* BG (UniProt: U3MZH0), and *Aspergillus fumigatus* BG (UniProt Q4WJJ3) were expressed in *Aspergillus oryzae* and purified as described previously [23]. The protein sequences were aligned and the sequence identity was calculated using Clustal Omega [26]. For the phylogenic analysis, amino acid sequences were selected from the CAZy database. Only sequences annotated with β-glucosidase activity (EC 3.2.1.21) with a PDB accession were selected. Alignment and phylogenetic analysis were performed in MEGAX [1]. The sequences were aligned using MUSCLE using default parameters. The phylogenetic tree was constructed using the maximum-likelihood method based on the Whelan and Goldman model (WAG + G) [2]. The log likelihood was − 8985.45. Purity was confirmed by the occurrence of a single band in SDS NuPAGE 4–12% BisTris gel (GE Healthcare) (Additional file 1: Fig. S6). *Aspergillus fumigatus* BG (*Af*BG) was subjected to further purification by size exclusion chromatography (SEC) on a HiLoad Sephadex 200 (26/600) gel filtration column (GE Healthcare) using a flow rate of 2 ml/min and 40 mM MES pH 6 with 100 mM NaCl as eluent. The purity of the *Af*BG sample was evaluated before and after the SEC step by two independent methods. First, mass spectrometry was used to analyze tryptic digests by filter-aided sample preparation (FASP) as described previously [14]. The second purity analysis was LabChip GXII microfluidic CE‐SDS electrophoresis (Perkin Elmer) with the Protein Express assay according to the instructions by the manufacturer. Prior to the CE-analysis, the protein was concentrated by trichloroacetic acid (TCA) precipitation. 10 µl 50% TCA was mixed with 40 µl protein. The mixture was kept on ice for 10 min after which the sample was centrifuged. The pellet was washed three times with 500 µl ice-cold acetone and resuspended in 8 M urea. The final enzyme load analyzed was about 30 µg.

### Activity measurements

All substrates were washed five times in MilliQ water in twice the volume and twice in assay buffer (50 mM acetate, pH 5.0; henceforth referred to as standard buffer) to remove traces of soluble saccharides. Substrate suspensions (225 µl) in standard buffer with varying loads of cellulose were transferred to 96-well microtiter plates. The final loads were in the ranges 0–95 g/l for Avicel and 0–5 g/l for PASC and BMCC. The reactions were started by the addition of 25 µl of BG stock to a final concentration of 100 nM. Control samples with 25 µl buffer instead of BG stock were included for all substrates and all substrate loads. The plates were stirred in an orbital mixer (Thermomixer C equipped with a ThermoTop, Eppendorf) at 1100 rpm and the desired temperature for 1 h. Subsequently, the plates were centrifuged for 3 min at 3500 rpm and 50 µl supernatant was transferred to 96-well PCR plates and 20 µl buffer was added to each sample, except the control samples. Control samples were added 20 µl BG stock to a final concentration of 100 nM and also incubated for 1 h. The concentration of soluble reducing sugars was quantified by the para-hydroxybenzoic acid hydrazide assay (PAHBAH). Specifically, 85 µl 15 g/l PAHBAH (p-hydroxybenzoic acid hydrazide) dissolved in 0.18 M potassium sodium tartrate and 0.5 M NaOH was added to each sample. The PCR plates were then incubated in a PCR instrument (Bio-Rad T100) at 95 ℃ for 5 min followed by 20 ℃ for 5 min. Hereafter 100 µl of each sample was transferred to a 96-well microtiter plate and the absorption at 405 nm was measured in a plate reader (Molecular Devices SpectraMax i3). The concentration of soluble reducing sugars was quantified based on standards with 0–1 mM glucose. All experiments were carried out in triplicates.

### High-performance anion exchange chromatography with pulsed amperometric detection (HPAEC)

Samples of 100 nM BG were incubated with 50 g/l Avicel in standard buffer to a final volume of 1 ml. The samples were stirred in an orbital mixer (Thermomixer C equipped with a ThermoTop, Eppendorf) at 1100 rpm at 50 °C 1 h. Subsequently, the reactions were quenched by the addition of 1 ml sodium hydroxide to a final concentration of 0.1 M NaOH. HPAEC was conducted using an ICS5000 system (Dionex). 25 µl of sample was injected on a CarboPac PA10 4 × 250 mm analytical column (Dionex) with an AminoTrap 4 × 50 mm column (Dionex) kept at 25 °C. The solutes were eluted at 1 ml/min with initial conditions set to 0.08 M NaOH and 0.025 M sodium acetate for 5 min. A linear gradient was applied to reach 0.19 M NaOH and 0.425 M sodium acetate after 5.5 min. Hereafter a linear gradient was applied to reach initial conditions after 6 min and these conditions were kept for 4 min.

### Re-start experiment

Samples of varying loads of Avicel were prepared as described above and added *Af*BG stock to a final concentration of 100 nM *Af*BG. The samples were allowed to react for 17 h at 25 °C with mixing at 1100 rpm as described above. Hereafter the plates were centrifuged and the supernatant was discarded. The pellet was washed five times with 250 µl standard buffer. The reaction was then re-started by the addition of 100 nM *Af*BG to the washed pellet. Finally, the release of reducing sugars was measured by the PAHBAH method after 1 h at 25 °C in the thermomixer using the same procedure as above.

### Adsorption measurements

Samples of Avicel (varying loads from 0 to 90 g/l) were incubated with 100 nM *Af*BG in a final volume of 250 µl in 96-well microtiter plates for 1 h. at 25 °C set to 1100 rpm in a thermomixer as described above. Subsequently, the plates were centrifuged for 3 min at 3500 rpm and 100 µl supernatant was transferred to black 96-well microtiter plates and 50 µl buffer was added to each well. The concentration of free enzyme was determined based on intrinsic fluorescence at 340 nm determined in a plate reader (Molecular Devices SpectraMax i3) using an excitation wavelength of 280 nm.

## Supplementary information

**Additional file 1: Figure S1.** Phylogenetic tree. **Figure S2.** Product profile. **Figure S3.** Re-start experiment. **Figure S4.** LabChip GXII microfluidic CE-SDS electrophoresis. **Figure S5.** Specific rate of *Af*BG before and after additional purification. **Figure S6.** SDS-gel. **Table S1.** Percent Identity Matrix. **Table S2.** FASP-MS.

## Data Availability

All data generated or analyzed during this study are included in this published article [and its additional files].

## Abbreviations

BG: β-Glucosidase
GH3: Glucoside hydrolase family 3
CBH: Cellobiohydrolase
EG: Endoglucanase
COS: Cellooligosaccharide
PASC: Phosphoric acid swollen cellulose
BMCC: Bacterial microcrystalline cellulose
DP: Degree of polymerization
SEC: Size exclusion chromatography
FASP-MS: Filter-aided sample preparation for mass spectrometry
PAHBAH: Para-hydroxybenzoic acid hydrazide
