# Supplementary material for: Activity of fungal β-glucosidases on cellulose
Source: Biotechnol Biofuels. 2020 Jul 10;13:121. doi: 10.1186/s13068-020-01762-4 (PMC7350674; doi:10.1186/s13068-020-01762-4)
Supplement: Supplementary file 1 — Additional file 1: Figure S1. Phylogenetic tree. Figure S2. Product profile. Figure S3. Re-start experiment. Figure S4. LabChip GXII microfluidic CE-SDS electrophoresis. Figure S5. Specific rate of AfBG before and after additional purification. Figure S6. SDS-gel. Table S1. Percent Identity Matrix. Table S2. FASP-MS. [file 13068_2020_1762_MOESM1_ESM.pdf]

Additional File 1

Activity of Fungal β-glucosidases on Cellulose

Malene B. Keller[1,2], Trine H. Sørensen[2,3], Kristian B. R. M. Krogh[3], Mark Wogulis[4], Kim Borch[3] and Peter Westh[5]

1. Department of Geosciences and Natural Resource Management, University of Copenhagen, 23 Rolighedsvej, DK-1958, Denmark
2. Department of Science and Environment, 1 Universitetsvej, DK-4000 Roskilde University, Denmark
3. Novozymes A/S, 2 Biologiens Vej, DK-2800 Kgs. Lyngby, Denmark
4. Novozymes Ltd, 1445 Drew Ave, Davis, CA 95618, USA
5. Department of Biotechnology and Biomedicine, Technical University of Denmark, 221 Søltofts Plads, DK-2800 Kgs. Lyngby, Denmark

Table S1 Percent Identity Matrix of *Aspergillus fumigatus* BG, *Aspergillus nidulans* BG, *Penicillum oxalicum* BG, and *Magnaporthe grisea* BG.

	Aspergillus nidulans GH3	*Magnaporthe grisea* GH3	*Penicillium oxalicum GH3*	*Aspergillus fumigatus* GH3
Aspergillus nidulans GH3	100.00	49.93	43.21	48.56
Magnaporthe grisea GH3		100.00	53.06	59.16
Penicillium oxalicum GH3			100.00	68.31
Aspergillus fumigatus GH3				100.00

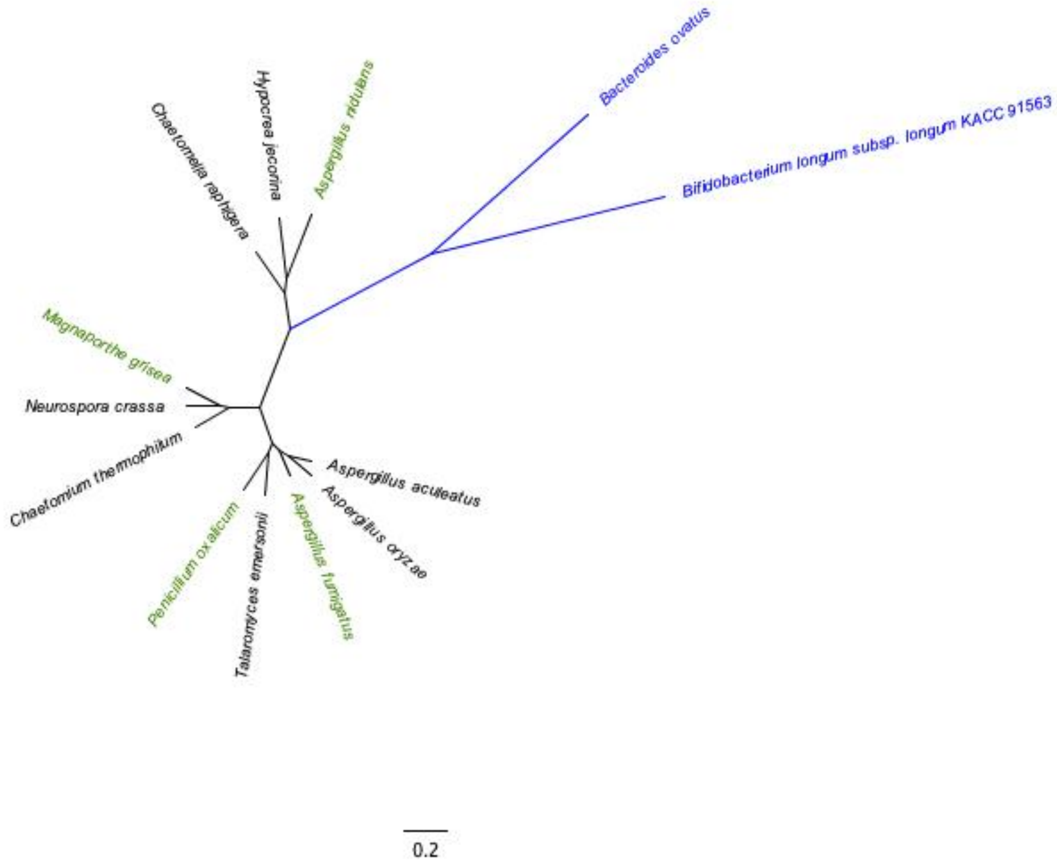

Figure S1 Phylogenetic tree of GH3. The sequences were selected from the CAZy database. Only characterized enzymes with annotated with β-glucosidase activity (EC 3.2.1.21), were selected. A multiple sequence alignment was performed using MUSCLE. Alignment and phylogenetic analysis were performed in MEGAX. The phylogenetic tree was constructed using the Maximum-Likelihood method based on the Whelan and Goldman Model. The tree with the highest log likelihood is shown (-8985.45). Sequences of bacterial origin are shown in blue lines. The scale bar is 0.2 amino acid substitutions per site. Sequences of fungal origin (all Ascomycota) are shown in black lines. The sequences characterized in this study are shown in green font. The sequences are named according to the host organism.

Uniprot accession numbers are: *Bacteroides ovatus (*A7LXU3), *Bifidobacterium longum subsp. longum* KACC 91563 (F8ATF7), *Aspergillus aculeatus (*P48825), *Aspergillus aculeatus (*P48825), *Aspergillus oryzae* (Q2UUD6), *Chaetomella raphigera* (A0A068FT77), *Chaetomium thermophilum* (A6YRT4), *Neurospora crassa (*Q7RWP2), *Hypocrea jecorina* (Q12715), *Talaromyces emersonii* (Q8TGI8*), Aspergillus nidulans* (A0A2T5LVU4), *Magnaporthe grisea* (L7J2B9), *Penicillium oxalicum* (U3MZH0), *Aspergillus fumigatus* (Q4WJJ).

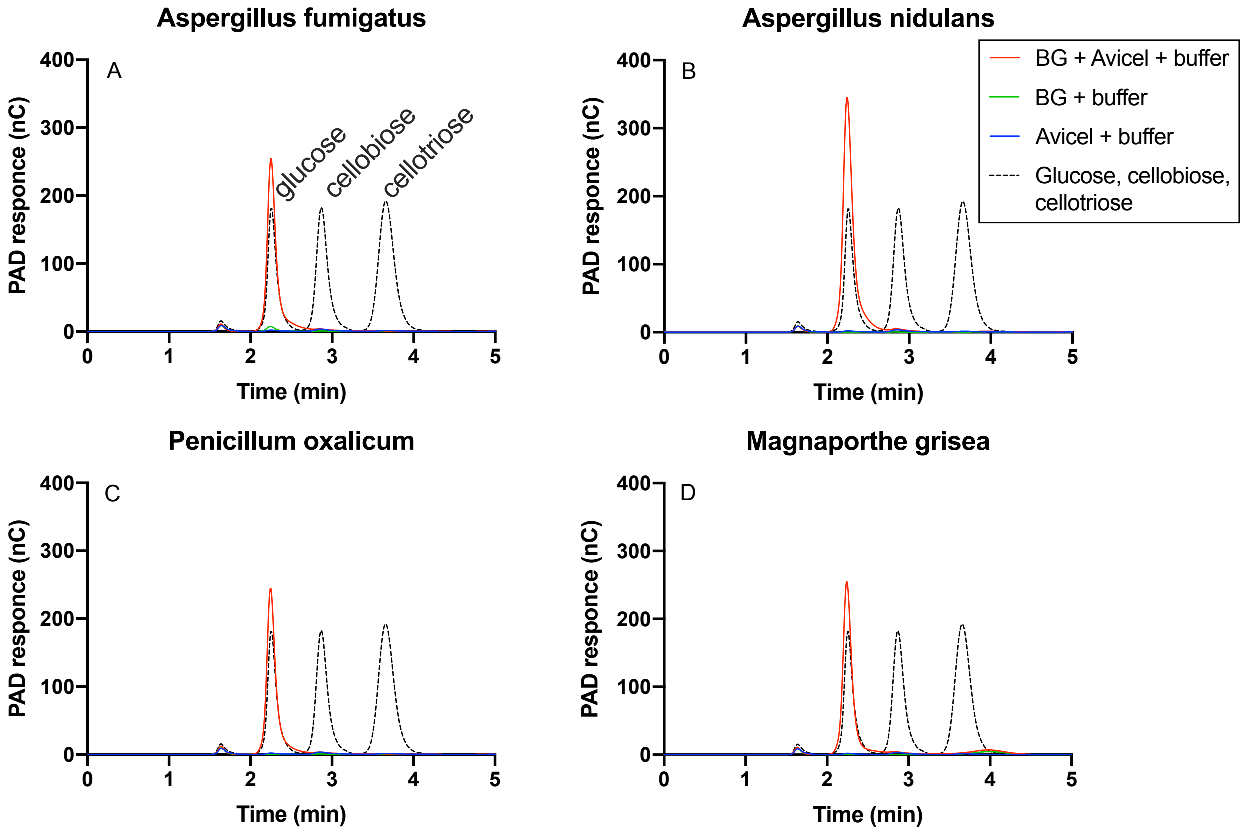

Figure S2 Product profile of the BGs. The product profile of *Aspergillus fumigatus* BG (A), *Aspergillus nidulans* BG (B), *Penicillum oxalicum* BG (C), *Magnaporthe grisea* BG (D). Chromatograms of the products of each BG on Avicel (red). Control experiments with BG and buffer (green) and Avicel and buffer (blue) are included. Standards containing glucose, cellobiose, and cellotriose are included (dotted lines). The elution times of the standards are indicated in A.

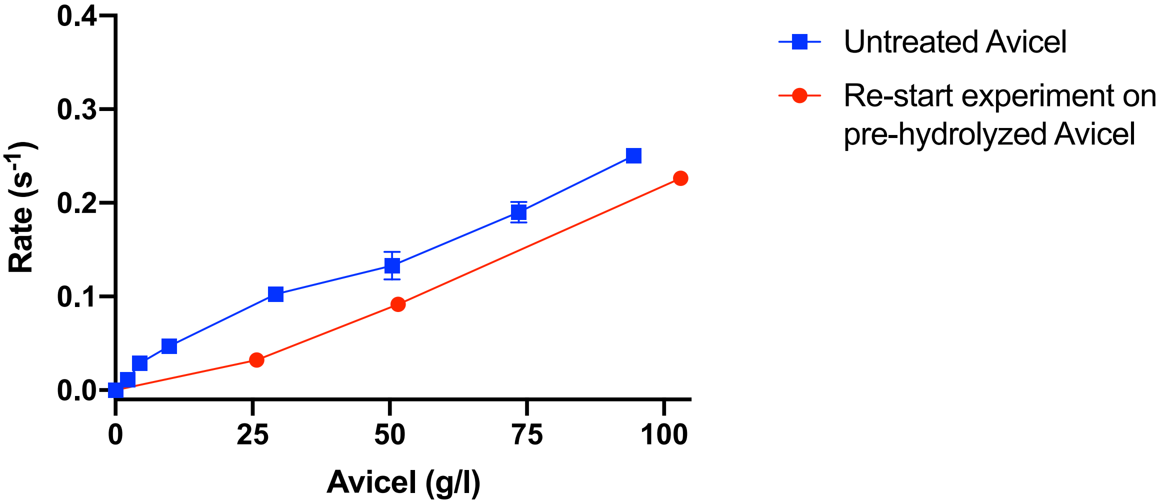

Figure S3 Re-start experiment. The specific rate of A. fumigatus BG plotted against substrate load on untreated Avicel (blue squares) and on pre-hydrolyzed Avicel (red circles). The pre-hydrolyzed substrate was incubated with A. fumigatus GH3 for 17 hr. Hereafter the substrate was washed and treated with A. fumigatus again. Lines between each point is shown to guide the eye.

Table S2 Purity evaluation of the A. fumigatus BG sample by filter aided sample preparation for mass spectrometry (FASP-MS).

	**Description**	**Activity**
99.444%	Aspergillus fumigatus beta-glucosidase polypeptide	b-glucosidase
0.003%	Uncharacterized protein	No modules
0.001%	ORF, Putative antigenic cell wall protein	Hydrophobic surface binding protein A
0.001%	Uncharacterized protein	short chain dehydrogenase
0.024%	Uncharacterized protein	Adaptor complexes medium subunit family
0.010%	Uncharacterized protein	No modules
0.001%	Uncharacterized protein	FAD binding domain
0.196%	Uncharacterized protein	Ubiquitin 3 binding protein But2 C-terminal domain
0.008%	Uncharacterized protein	SPFH domain / Band 7 family
0.004%	Uncharacterized protein	Alpha/beta hydrolase family
0.003%	Uncharacterized protein	No modules
0.000%	Uncharacterized protein	FAD binding domain
0.086%	orf, secreted protein with EGF motif	No modules
0.028%	Uncharacterized protein	Gaa1-like, GPI transamidase component
0.004%	Uncharacterized protein	putative exo-Î²-1,3-glucanase
0.188%	Probable beta-glucosidase A	b-glucosidase

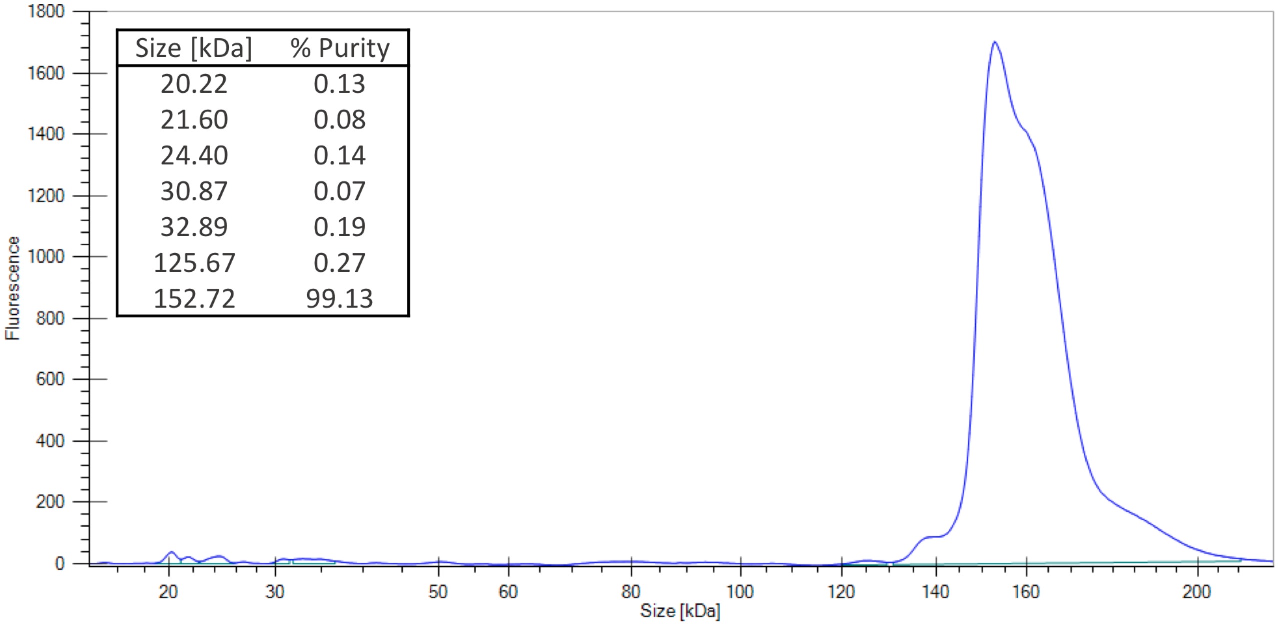

Figure S4 LabChip GXII microfluidic CE-SDS electrophoresis chromatogram of the Aspergillus fumigatus BG sample after additional purification. The insertion shows the purity of the detected peaks.

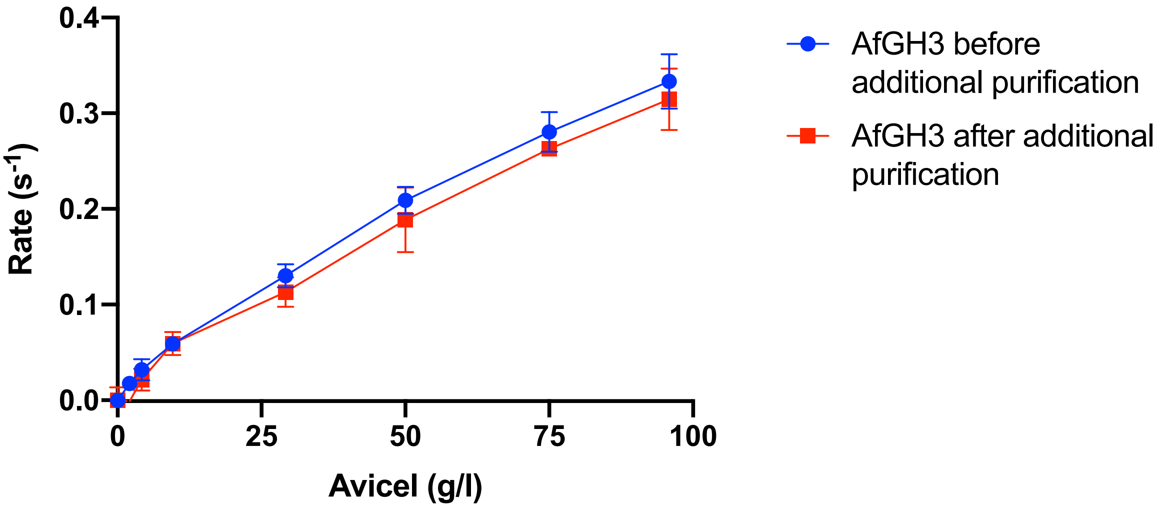

Figure S5 The specific rate of A. fumigatus BG plotted against Avicel load for A. fumigatus BG before the additional purification (blue circles) and after the additional purification (red sqaures). Lines between each point is shown to guide the eye.

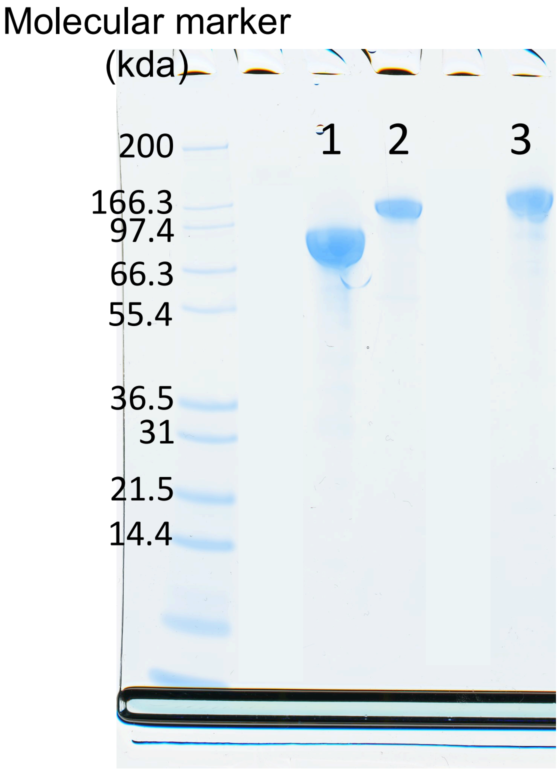

Figure S6 SDS-gel of BGs. The proteins were stained with InstantBlue protein stain, Cromassie Brilliant Blue (Expedeon). The amount of β-glucosidase on the gel was 2.5 µg. 1. *Aspergillus nidulans* BG, 2. *Magnaporthe grisea* BG, 3. *Penicillium oxalicum* BG.
